# Fast-Track Diagnostic Pathway for Lung Cancer Detection: Single-Center Experience

**DOI:** 10.3390/jcm14092915

**Published:** 2025-04-23

**Authors:** Valentina Tassi, Roland Peraj, Daina Pietraforte, Fabrizio Benedetti, Alessio Gili, Annalisa Guida, Cristina Zannori, Fabio Arcidiacono, Luisa Lo Conte, Benedetta Enrico, Linda Ricci, Roberto Cirocchi, Mark Ragusa

**Affiliations:** 1 Thoracic Surgery Unit, Santa Maria Hospital, University of Perugia, 05100 Terni, Italy; v.tassi@aospterni.it (V.T.); rolandperaj92@gmail.com (R.P.); daina.pietraforte@aospterni.it (D.P.); f.benedetti@aospterni.it (F.B.); m.ragusa@aospterni.it (M.R.); 2 Public Health Section, Department of Medicine and Surgery, University of Perugia, 06123 Perugia, Italy; a.gili@unilink.it; 3 Medical Oncology Unit, Santa Maria Hospital, 05100 Terni, Italy; a.guida@aospterni.it (A.G.); c.zannori@aospterni.it (C.Z.); 4 Radiation Oncology Centre, Santa Maria Hospital, 05100 Terni, Italy; f.arcidiacono@aospterni.it; 5 Nuclear Medicine Service, Santa Maria Hospital, 05100 Terni, Italy; l.loconte@aospterni.it; 6 Interventional Radiologist Unit, Santa Maria Hospital, 05100 Terni, Italy; b.enrico@aospterni.it; 7 Pathology Unit, Santa Maria Hospital, University of Perugia, 05100 Terni, Italy; l.ricci@aospterni.it; 8 Department of Medicine and Surgery, Santa Maria Hospital, University of Perugia, 05100 Terni, Italy

**Keywords:** lung cancer early diagnosis, diagnostic pathways, time to biopsy, time to ^18FDG^PET-CT, multidisciplinary team, patients’ satisfaction

## Abstract

**Objectives**: Despite continuous advances in diagnosis, such as the “Two week wait” policy for hospital specialist referral and fast-track diagnostic pathways, lung cancers are detected mostly at advanced stages. Our aim was to evaluate the fast-track diagnostic pathway in a tertiary hospital. **Methods**: Between March and September 2022, 114 consecutive patients with respiratory symptoms or radiology suspicions of lung cancer were referred to our “Pulmonary Point” outpatient clinic. The time intervals to take in the charges and conduct biopsy and ^18FDG^PET-CT were prospectively collected. Furthermore, the patients’ experiences were evaluated by means of a six-item questionnaire investigating the outpatient clinic environment and accessibility, the kindness and professional approach of the healthcare professionals, the psychological support provided and an overall evaluation. The data were compared with those of 79 patients observed in the Thoracic Surgery Ambulatory in the pre-COVID-19 pandemic period of March–September 2019 before the fast-track diagnostic pathway for lung cancer was established. **Results**: The patients were referred to the “Pulmonary Point” outpatient clinic by a General Practitioner in 44 cases (38.5%), by other Specialists in 56 (49.1%) and by an Emergency Department in 14 (12.2%). Among the 114 patients, 104 (91.2%) were visited within 3 working days. Biopsies (FNAB, EBUS, bronchoscopy or surgical) were performed at a median period of 18 days (IQR: 9–26), and ^18FDG^PET-CT was carried out at a median period of 16 days (IQR: 7–25). The patients referred to the Thoracic Surgery Ambulatory in the period of March–September 2019 were characterized by longer times to biopsy [26 days (IQR: 12–54), *p* < 0.001] and to ^18FDG^PET-CT [25 days (IQR: 15–38), *p* = 0.003]. The patients referred in 2022 reported higher scores in the clinic environment (*p* < 0.001), psychological support provided (*p* < 0.001) and overall evaluation (*p* = 0.02) domains of the questionnaire. **Conclusions**: The establishment of a dedicated diagnostic pathway improves time to diagnosis and patients’ satisfaction.

## 1. Introduction

Lung cancer is the second most commonly diagnosed malignant tumor and the first most common cause of neoplastic death worldwide. The most common histological type is non-small-cell lung cancer (NSCLC), accounting for 85% of all lung cancer cases [1,2]. About 30% of new NSCLC cases are diagnosed at a locally advanced stage, which encompasses a wide group of different clinical scenarios with a heterogeneous spectrum of therapeutic options [3,4,5]. Furthermore, with the widespread application of computed tomography (CT), the number of patients with incidentally found pulmonary nodules has progressively increased. Accurate lung cancer diagnosis and staging require various imaging studies, ranging from Positron Emission Tomography (^18FDG^PET) to brain Magnetic Resonance (MR), to rule out unsuspected metastatic disease and reduce non-curative surgical resections [6,7]. Moreover, minimally invasive needle techniques, such as endobronchial ultrasound-guided needle aspiration (EBUS), have now been largely adopted to assess the mediastinal stage of the disease [8,9,10]. In a multidisciplinary setting, the work-up of patients with suspected lung cancer requires prompt access to these tests in order to minimize delays [11,12,13]. Given that the timeliness of lung cancer care is an important quality indicator, efforts have been made to coordinate and accelerate the diagnostic pathways worldwide; in the last twenty years, the National Institute for Health and Clinical Excellence and other authoritative societies [14,15,16] have recommended the establishment of rapid-access clinics to deliver timely care, as well as the “Two week wait” policy promoted in the UK [17,18,19]. In our Hospital, the Azienda Ospedaliera “Santa Maria” at Terni, Italy, an original secondary-level ambulatory activity addressed to patients presenting with generic respiratory symptoms such as cough, dyspnea and doubtful imaging of any type in traditional lung radiology was established. At the Pulmonary Point ambulatory (Pu.Po. is an Italian nickname for “kid” and stands for “Pulmonary Point”), each patient was evaluated together by a Thoracic Surgeon and a Pneumologist and was immediately addressed in the fast-track diagnostic–therapeutic pathway in case of an oncological suspicion. The aim of this study was to assess the impact of this model of care on the timeliness of lung cancer diagnosis and staging.

## 2. Materials and Methods

**Figure 1:**
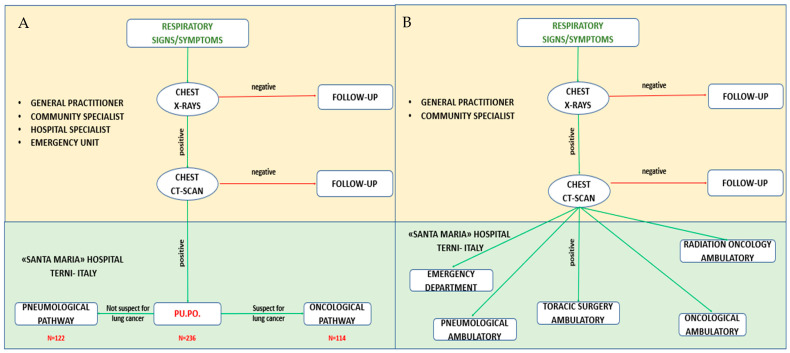
(**A**). How patients with suspected lung cancer may access the Pulmonary Point outpatient clinic established in 2022 and start the fast-track diagnostic pathway. Pu.Po: Pulmonary Point outpatient clinic. (**B**). How patients with suspected lung cancer were referred to Hospital Specialists before the Pulmonary Point outpatient clinic access and the fast-track diagnostic pathway started.

**Figure 2:**
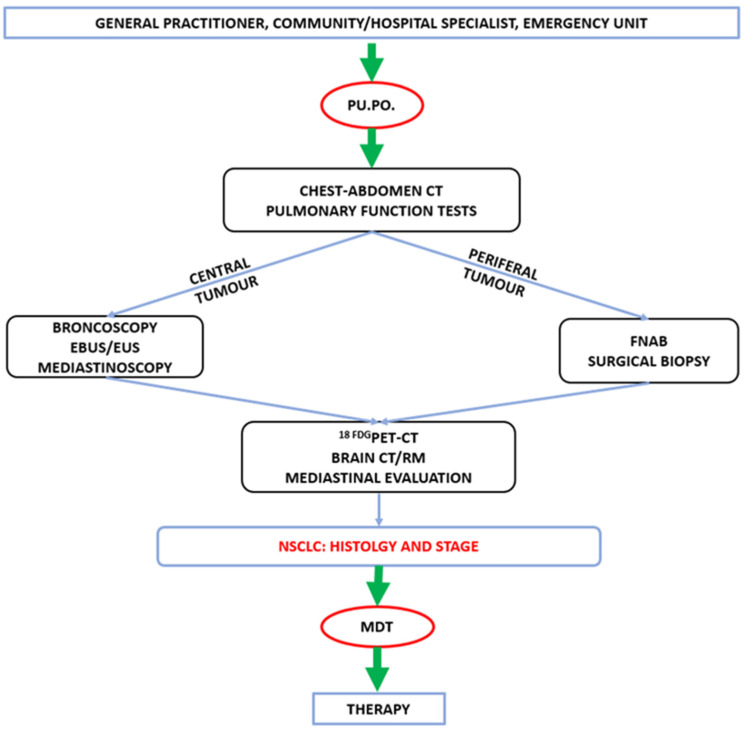
Fast-track diagnostic pathway for lung cancer detection. Pu.Po: Pulmonary Point outpatient clinic; EBUS: endobronchial ultrasound; EUS: Endoscopic Ultrasound; FNAB: Fine-Needle Aspiration Biopsy; NSCLC: non-small-cell lung cancer; MDT: multidisciplinary team.

Since February 2022, the Azienda Ospedaliera “Santa Maria” at Terni in Italy started a diagnostic–therapeutic route for patients with suspected lung cancer together with the dedicated Pu.Po. ambulatory. The Azienda Ospedaliera “Santa Maria” is one of the two tertiary hospitals of our region, serving a population of approximately 900,000 people. The hospital has the personnel, skills and equipment for a complete diagnostic work-up including a chest CT scan, ^18FDG^PET-CT, MR, bronchoscopy, EBUS and Endoscopic Ultrasound (EUS), as well as percutaneous biopsies and surgical excision biopsies. Furthermore, the Pathology department provides the know-how for the molecular profiling of NSCLC. With regard to the therapeutic phase, the hospital has the possibility of providing state-of-the-art techniques and technologies in the chemotherapy, radiation therapy and surgical settings, including stereotactic ablative radiotherapy (SABR), radiofrequency ablation and minimally invasive thoracic surgery, with both video-assisted and robotic-assisted technology. The diagnostic pathway is summarized in Figure 1 and Figure 2. Briefly, patients with signs and symptoms suggestive of lung cancer or positive chest CT scans were enrolled. The General Practitioner, the Hospital or Ambulatory Specialist and the Emergency Department referred each patient to the “Pu.Po.” ambulatory by phone call, email or direct contact. Each appointment was scheduled within a working day, and each patient was visited within 3 working days. A Thoracic Surgeon and a Pulmonologist visited each patient together and referred him or her to a pneumological route or an oncological fast-track diagnostic–therapeutic pathway according to the findings of the check-up (Figure 1).

In the oncological setting, each patient was driven along the diagnostic–therapeutic route by the referral Specialist and a dedicated nurse (Figure 2). All cases were discussed by the local multidisciplinary team, involving a Medical Oncologist, a Pneumologist, a Radiologist, a Nuclear Medicine physician, a Radiation Therapist, a Pathologist and a Thoracic Surgeon, both in the diagnostic and the therapeutic phase.

In this study, data regarding the patients consecutively referred to our “Pu.Po.” outpatient clinic between March and December 2022 were prospectively collected. All invasive and radiological procedures aimed at providing diagnosis and staging were recorded. These included ^18FDG^PET-CT, bronchoscopies, EBUS and mediastinoscopies, as well as procedures aimed at sampling peripheral lung nodules, like Fine-Needle Aspiration Biopsies (FNABs) or surgical biopsies. The Performance Statuses of the patients were assessed according to the ECOG (Eastern Cooperative Oncology Group) scale. The 8th Edition of the TNM staging system was applied to define the stages of the disease. The time intervals from the “Pu.Po.” clinic visit to ^18FDG^PET-CT and from the “Pu.Po.” clinic visit to biopsy were calculated. Furthermore, the patients’ experiences were assessed by means of a 6-item questionnaire investigating the outpatient clinic environment and accessibility, the kindness and professional approach of the healthcare professionals, the psychological support provided and overall satisfaction. A dedicated nurse administered the 5-point Likert-scale questionnaire within a month after the end of the diagnostic–therapeutic pathway. The scores for each domain ranged from 0 (very dissatisfied) to 4 (very satisfied). The Thoracic Surgeon screened the patients as being suitable for questionnaire administration; they were required to be at least 18 years of age, to be able to read Italian and to complete a questionnaire. Patients who had dementia and so were not suitable to attend the interview were excluded from this study. The data were compared with those of 80 patients observed at the Thoracic Surgery Ambulatory in the pre-COVID-19 pandemic period, March–December 2019, before the fast-track diagnostic pathway for lung cancer was launched.

### Statistical Analysis

The present study was performed in accordance with the ethical standards of the Helsinki Declaration of the World Medical Association. The local institutional review board approved using the database from the thoracic surgery division for research purposes. All patient information, including illustrations, was anonymized. The data are represented as the medians and interquartile ranges (IQRs) for continuous variables and as n (%) for categorical variables. The χ2 test or Fisher’s test (expected number: less than 5) and the Mann–Whitney test were used to analyze the categorical and continuous variables, respectively. *p*-values of < 0.05 were considered significant. Univariate and multivariate linear regression approaches were used to confirm the differences in times to biopsy and times to ^18FDG^PET-CT considering the ages, genders, ECOG Performance Statuses and lung cancer stages of the patients involved. The data were analyzed using SPSS (version 15.0) (SPSS, Inc., Chicago, IL, USA).

## 3. Results

In the period of March–December 2022, 236 patients were referred to the Pu.Po. outpatient clinic of the Azienda Ospedaliera “Santa Maria” at Terni and visited by a Thoracic Surgeon and a Pneumologist. Clinical histories and chest CT scan images provided suspicion of lung cancer in 114 patients who were addressed to the fast-track diagnostic–therapeutic route; the remaining 122 cases, with low probabilities of cancer, were scheduled for a radiological follow-up program or further pneumological examinations.

The study population included 68 males (59.6%) and 46 females (40.3%), with a median age of 71 years (IQR: 73–76 years). A total of 98 (85.9%) were residents in the Terni area. The patients were referred to the “Pu.Po.” outpatient clinic by a General Practitioner in 44 cases (38.5%), by a Hospital/Community Specialist in 56 (49.1%) and by an Emergency Department in 14 (12.2%).

Regarding the visit scheduling, 55 (48.2%) were assigned by the regional appointment system, 29 (25.4%) contacted the ambulatory by phone or email and 30 (26.3%) came personally. Among the 114 patients, 104 (91.2%) were visited within 3 working days. In total, 91 (79.9%) patients were ECOG 0–1, and 23 (20.1%) were ECOG 2–3. Pathological diagnosis was obtained by means of pulmonary resection in 28 cases, FNAB in 39, bronchial biopsy in 23, EBUS in 15 and pleural biopsy in 9. Biopsy (FNAB, EBUS, bronchoscopy or surgical) was performed at a median period of 18 days (IQR: 9–26), and ^18FDG^PET-CT was carried out at a median period of 16 days (IQR: 7–25). The lung cancer stage distribution was as follows: 49 (42.9%) at stage I, 19 (16.6%) at stage II, 23 (20.1%) at stage III and 23 (20.1%) at stage IV. At the completion of the diagnostic phase, 106 cases (92.93%) were discussed by the multidisciplinary team.

The control group consisted of 79 patients referred to the Thoracic Surgery Ambulatory in the period of March–December 2019. There were 39 men (49.3%) and 40 women (50.6%), with a median age of 67 years (IQR: 67–74). A total of 60 (75.9%) lived in the Terni area. The patients were referred to the “Pu.Po.” outpatient clinic by a General Practitioner in 38 cases (48.1%), by a Hospital/Community Specialist in 37 (46.8%) and by an Emergency Department in 4 (5.1%).

All patients were scheduled by the regional appointment system because in that period, the fast-track pathway had not been started yet. In total, 64 (81.1%) patients were ECOG 0–1 and 15 (18.9%) were ECOG 2–3. Pathological diagnosis was obtained by means of pulmonary resection in 20 cases, FNAB in 32, bronchial biopsy in 12, mediastinoscopy in 2, EBUS in 5 and pleural biopsy in 7. The median time to biopsy was 26 days (IQR: 12–54), and the median time to ^18FDG^PET-CT was 25 days (IQR: 15–38). According to the 8th edition of TNM, the lung cancer stage distribution was as follows: 41 (51.8%) at stage I, 16 (20.2%) at stage II, 10 (12.6%) at stage III and 12 (15.1%) at stage IV. Comparing the two groups of patients (Table 1), we found that they were homogeneous for gender distribution (*p* = 0.23), ECOG Performance Status (*p* = 0.95) and lung cancer stage (*p* = 0.24), but the patients observed in 2019 were significantly younger than those observed in 2022 (*p* = 0.04). Furthermore, the patients referred to the Thoracic Surgery Ambulatory in 2019 were characterized by longer times to biopsy (*p* < 0.001) and to ^18FDG^PET-CT (*p* = 0.003). In the multivariate analysis, the time to biopsy was only influenced by the period of this study (2022 vs. 2019, *p* < 0.001; 95% CI: 9.17–24.88) and not by age (*p* = 0.32), gender (*p* = 0.3), ECOG Performance Status (*p* = 0.67) or lung cancer stage (*p* = 0.6). Similarly, the period of this study (2022 vs. 2019, *p* = 0.002; 95% CI: 2.96–12.89) and the gender distribution (male vs. female, *p* = 0.03; 95% CI: −12.21–12.51) had an effect on the time to ^18FDG^PET-TC; age (*p* = 0.635), ECOG Performance Status (*p* = 0.861) and lung cancer stage (*p* = 0.745) did not.

**Figure 3:**
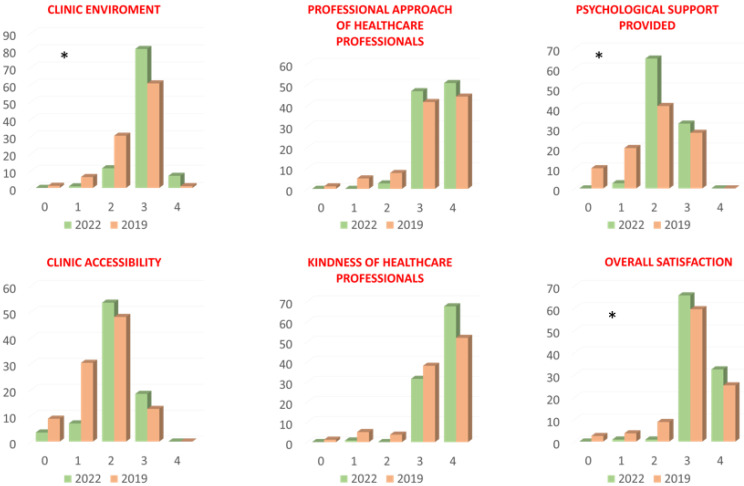
Results of the Satisfaction Questionnaire investigating the outpatient clinic environment and accessibility, the kindness and professional approach of the healthcare professionals, the psychological support provided and overall satisfaction in the two groups of patients observed in 2022 (green bar) and in 2019 (orange bar). The scores for each domain ranged from 0 (very dissatisfied) to 4 (very satisfied). * *p* < 0.05.

With regard to the Satisfaction Questionnaire, all patients completely understood the questions, as all of them had completed at least first-grade school instruction, as detailed in Table 1. The patients referred in 2022 reported higher scores in the clinic environment (*p* < 0.001), psychological support provided (*p* < 0.001) and overall satisfaction (*p* = 0.02) domains. The remaining domains (clinic accessibility, kindness and professional approach of healthcare professionals) did not show statistically significant variations between the two groups (Figure 3 and Table 2).

## 4. Discussion

We conducted a comprehensive analysis on the timeliness of care for patients with suspected NSCLC referred to our new fast-track diagnostic clinic called “Pulmonary Point” (Pu.Po.). This study focused on the waiting times for diagnosis achieved in the new setting compared with those measured before the institution of the dedicated pathway. The strengths of our program included early specialist consultation, nurse-led care coordination and a multidisciplinary approach. A major finding was that 91% of the patients (104/114) were visited by the Thoracic Surgeon and the Pneumologist within 3 working days. Furthermore, the time intervals to biopsy and to ^18FDG^PET-CT were significantly shorter when performed according to the fast-track diagnostic route (*p* < 0.001 and *p* = 0.003, respectively) instead of the standard one. Although the data on the time to the treatment intervals are not available for this study, the median times to the biopsy and to the ^18FDG^PET-CT were 18 and 16 days, respectively, both within the recommended diagnostic wait time of 2 months [16]. Similarly, thanks to the establishment of the Pu.Po. ambulatory, the patients were referred for specialist consultation well within the 7-day interval suggested by the BTS Guidelines [14]. In the English literature, various predictors of increased time to diagnosis and treatment have been reported: those related to healthcare system factors (care at an academic center, necessity of patient transport between facilities) and those that are patient-related (race, education, prior history of cancer, comorbidities and age) [20,21,22,23]. In a population-based series, the median wait times were significantly shorter for patients with stage III and IV disease, poor Performance Status and small-cell histology, reflecting the phenomenon of physicians acting “quicker on sicker patients” [24,25]. In our experience, more than 50% of patients were able to schedule their own appointment at Pu.Po. ambulatory by phone, email or coming personally; we postulate that such simplification of the scheduling mechanism is the reason why over 90% of patients were visited in an appreciably short time. This observation was further confirmed considering that the patients visited in 2022 were significantly older than their 2019 counterparts (*p* = 0.04). It is worth underscoring that a nurse navigator carried out a triage aimed at identifying urgent cases, facilitated the enrollment process and played an important role in developing personal relationships with patients and in communicating the plans developed by the thoracic specialists at the weekly multidisciplinary meetings [26,27]. The importance of such meetings is pivotal: 93% of our patients (106/114) were discussed in such multidisciplinary settings in order to define the most appropriate diagnostic and therapeutic work-ups, reducing inappropriate investigations and thus diagnostic delay. It has been demonstrated that multidisciplinary discussion improves patients’ overall survival due to a reduction in the number of advanced-stage cancer patients proceeding to surgery, a more accurate selection of patients and the benefits derived from the increased cumulative experience of different specialists. Moreover, the literature reports a higher rate of enrollment in clinical trials among patients who actually were seen at the clinic by multidisciplinary team members [11,28,29]. These findings have led us to recommend the management of all cancer patients by a multidisciplinary board at our institution, irrespective of staging, both in the diagnostic and in the therapeutic route.

At last, the perceived quality from the ambulatory patients’ point of view was higher in the 2022 case group. Those patients returned higher scores in the clinic environment (*p* < 0.001), psychological support provided (*p* < 0.001) and overall satisfaction (*p* = 0.02) domains. Although not specifically investigated, a faster and more effective diagnostic “package”, along with the presence of a dedicated nurse, resulted in an increase in patients’ satisfaction [30,31].

The main limitations of our study are its single-center structure with high potential for institutional bias and the relatively small number of patients. Further studies, hopefully multicentric, are needed to confirm our data and to investigate the impact of dedicated pathways on the timeliness of lung cancer diagnosis and staging.

## 5. Conclusions

In conclusion, outside of a specifically designed study, the raw literature data do not demonstrate a favorable impact of waiting-time reduction on survival in the lung cancer setting. This study also did not allow us to draw any conclusions on the long-term outcomes of lung cancer treatment, but it suggests that the establishment of a dedicated diagnostic pathway may improve time to diagnosis and patients’ satisfaction.

## Figures and Tables

**Table 1:** Demographics, visit scheduling and times to diagnosis of 114 patients observed at the “Pulmonary Point” Outpatient Ambulatory in 2022 and of 79 patients visited at the Thoracic Surgery Ambulatory in 2019.

	Group 2022	Group 2019
	(N = 114)	(N = 79)
**Gender**		
Males	68 (59.6%)	39 (49.3%)
Females	46 (40.4%)	40 (50.6%)
**Age **		
Median (IQR)	71 years (IQR: 73–76)	67 years (IQR: 67–74)
**Education degree**		
Primary school	13 (11.4%)	6 (7.6%)
Secondary school	101 (88.6%)	73 (92.4%)
**Residence**		
Terni area	98 (86%)	60 (76%)
Extra-Terni area	16 (14%)	19 (24%)
**Referring physician**		
General Practitioner	44 (38.6%)	38 (48.1%)
Hospital/Community Specialist	56 (49.1%)	37 (46.8%)
Emergency Department	14 (12.2%)	4 (5%)
**Visit scheduling**		
Regional appointment system	55 (48.2%)	79 (100%)
Phone or email	29 (25.4%)	
Personally	30 (26.3%)	
**Performance status**		
0–1	91 (79.9%)	64 (81.1%)
2–3	23 (20.1%)	15 (18.9%)
**Stage (TNM 8th Edition)**		
I	49 (42.9%)	41 (51.8%)
II	19 (16.6%)	16 (20.2%)
III	23 (20.1%)	10 (12.6%)
IV	23 (20.1%)	12 (15.1%)
**Time to 18FDGPET-TC**		
Median (IQR)	16 days (IQR: 7–25)	25 days (IQR: 15–38)
**Time to biopsy**		
Median (IQR)	18 days (IQR: 9–26)	26 days (IQR: 12–54)

**Table 2:** Results of the Satisfaction Questionnaire investigating the outpatient clinic environment and accessibility, the kindness and professional approach of the healthcare professionals, the psychological support provided and overall satisfaction in the two groups of patients observed in 2022 and in 2019.

	Group 2022 (N = 114)	Group 2019 (N = 79)	*p*-Value
**Clinic environment**			*p* < 0.001
Median (IQR)	3 (3–3)	3 (2–3)	
Mean ± SD	2.93 ± 0.46	2.54 ± 0.69	
**Clinic accessibility**			*p* = 0.06
Median (IQR)	2 (1–2)	2 (1–2)	
Mean ± SD	1.86 ± 0.74	1.64 ± 0.81	
**Kindness of healthcare professionals**			*p* = 0.4
Median (IQR)	4 (3–4)	4 (3–4)	
Mean ± SD	3.65 ± 0.52	3.55 ± 0.87	
**Professional approach of healthcare professionals**			*p* = 0.1
Median (IQR)	4 (3–4)	3 (3–4)	
Mean ± SD	3.48 ± 0.55	3.22 ± 0.89	
**Psychological support provided**			*p* < 0.001
Median (IQR)	2 (1–2)	1 (0–2)	
Mean ± SD	1.31 ± 0.83	0.89 ± 0.85	
**Overall satisfaction**			*p* = 0.02
Median (IQR)	3 (3–4)	3 (3–4)	
Mean ± SD	3.29 ± 0.53	3.01 ± 0.85	

## Data Availability

The data are only available on request due to privacy restrictions.

## Abbreviations

The following abbreviations are used in this manuscript:

Pu.Po	Pulmonary Point outpatient clinic
EBUS	Endobronchial ultrasound
EUS	Endoscopic Ultrasound
FNAB	Fine-Needle Aspiration Biopsy
NSCLC	non-small-cell lung cancer
MDT	Multidisciplinary team
